# Anti-inflammatory effects of Kupffer cells through α7-nicotinic acetylcholine receptors

**DOI:** 10.1186/cc11945

**Published:** 2013-03-19

**Authors:** Y Li, X Shi

**Affiliations:** 1 Changzheng Hospital, Second Military Medical University, Shanghai, China

## Introduction

Nicotine exerts anti-inflammatory effects in several cell types. α7-nicotinic acetylcholine receptor (α7-nAChR), which has high permeability to calcium, is believed to contribute significantly to nicotinic anti-inflammatory effects. However, the molecular mechanism is largely unknown. Kupffer cells in the liver play an important role in inflammatory response to pathogens invading, but whether there is α7-nAChR expression in Kupffer cells or cholinergic anti-inflammatory pathway involved in this process remains unclear.

## Methods

(1) Kupffer cells, isolated by collagenase digestion and differential centrifugation from mice and labeled with FITC-aBGT, were observed under laser scanning confocal microscope to test the expression of α7-nAChR. Protein level was also tested by western blotting, with RAW264.7 as positive control; (2) 100 nM LPS was given to Kupffer cells, with or without 1 mM nicotine. TNFα, IL-10 and HMGB-1 were tested at 4 hours, 12 hours or 24 hours, respectively; (3) 100 BALB/c mice were randomly divided into four group: Group I (only lethal dose of LPS was given), Group II (nicotine and LPS were given), Group III (LPS, nicotine and GdCl3 were given), and Group IV (LPS and nicotine were given and the left cervical vagus nerve was cut off). The mortality of mice was observed for 72 hours.

## Results

(1) Expression of α7-nAChR in Kupffer cells was confirmed by confocal microscope and western blotting; (2) after nicotine was administered, the level of TNFα and HMGB-1 increased and the level of IL-10 decreased. Given left cervical vagus nerve cut off or aBGT, the effect of nicotine was weakened; (3) Group I had the highest mortality rate, while in Group II nicotine did reduce the mortality rate dramatically. After the left cervical vagus nerve was cut off or aBGT was given, the effects of nicotine were weakened. Difference for the mortality rate between Group III and Group IV was not significant.

## Conclusion

Kupffer cells played a crucial rule in modulating inflammation and the anti-inflammatory effect of nicotine was partially weakened after left cervical vagus nerve cut off or aBGT was given. It was verified that left cervical vagus nerve was essential for the anti-inflammatory effect of nicotine and α7 acetylcholine receptors might play a critical role.
